# An international survey of contemporary practices towards fertility assessment and preservation in patients undergoing radical inguinal orchidectomy for testicular cancer

**DOI:** 10.1002/bco2.356

**Published:** 2024-04-26

**Authors:** Abi Kanthabalan, Anthony Emmanuel, Cameron Alexander, Nikita Bhatt, Vinson Chan, Odunayo Kalejaiye, Krishna Narahari, Veeru Kasivisvanathan, Majid Shabbir

**Affiliations:** ^1^ Department of Urology Worcestershire Royal Hospital Worcester UK; ^2^ Department of Urology Freeman Hospital Newcastle upon Tyne UK; ^3^ Department of Urology Royal Bolton Hospital Manchester UK; ^4^ Department of Urology Norfolk and Norwich University Hospital Norwich UK; ^5^ School of Medicine, Faculty of Medicine and Health University of Leeds Leeds UK; ^6^ Department of Urology University Hospitals Bristol and Weston NHS Foundation Trust Bristol UK; ^7^ Department of Urology Cardiff and Vale University Health Board Cardiff UK; ^8^ Division of Surgery and Interventional Science University College London London UK; ^9^ Department of Urology Guy's & St. Thomas' Hospital London UK; ^10^ Faculty of Life Sciences & Medicine King's College London

**Keywords:** radical inguinal orchidectomy, semen cryopreservation, survey, testicular cancer

## Abstract

**Objective:**

The study aims to assess current international clinician attitudes, practices and barriers towards fertility assessment and preservation in patients undergoing radical inguinal orchidectomy (RIO) for testicular cancer.

**Materials and methods:**

An international online survey of urologists and urologists in training who perform RIO for testicular cancer was developed by the British Association of Urological Surgeons (BAUS) Sections of Andrology and Oncology and the British Urology Researchers in Surgical Training (BURST). The recruitment process used social media and the emailing lists of national urological societies. Responses were collected between 10/02/2021 and 31/05/2021 and stored using password‐protected Research Electronic Data Capture (REDCap) database software. The primary outcome was the proportion of urologists who routinely offer semen cryopreservation prior to RIO. The study was reported according to the Checklist for Reporting Results of Internet E‐Surveys platform.

**Results:**

A total of 393 respondents took part in the online survey; of these, the majority were from the United Kingdom (65.9%), with the remaining international respondents (34.1%) from six different continents, which included 45 different countries. Of the respondents, 57.1% reported that they would routinely offer semen cryopreservation to all patients undergoing RIO for testicular cancer. In addition, 36.0% of urologists routinely performed pre‐operative semen analysis, and 22.1% routinely performed pre‐operative testicular serum hormone profile. Of the respondents, 14.4% performed expedited RIO within 48 h; 31.2% of respondents reported that they considered no delay to RIO to allow for semen cryopreservation to be acceptable.

**Conclusions:**

A significant proportion of international urologists do not offer pre‐operative fertility assessment and preservation in men undergoing RIO for testicular cancer. Surgery is performed in an expedited fashion within 1 week in the majority of patients. Urologists perceive there to be a lack of access and availability to fertility services, and that delay to RIO to allow for fertility preservation is often not acceptable.

## 1 INTRODUCTION

Testicular cancer is the most common cancer in young men with reproductive potential with peak incidence in men in their fourth decade of life.^1^ Up to 24% of testicular cancer patients are azoospermic and 50% are oligospermic prior to commencing treatment.^2^, ^3^ Defects in spermatogenesis have been observed in the unaffected, contralateral testis in 24% of men with testicular germ cell tumours.^4^ Radical inguinal orchidectomy (RIO) can further compromise sperm count, with previous studies demonstrating impaired post‐operative semen parameters in 85% of cases, and the development of azoospermia in 9%.^5^, ^6^, ^7^ Chemotherapy and radiation treatment (RT) can further impair conception by up to 30% and where spermatogenesis does recover after chemotherapy, this may take up to 4 years.^3^

Despite significant improvements in long‐term testicular cancer survival outcomes, it remains unclear if current international practice has changed to optimise fertility outcomes. International guidelines recommend that all men should be offered sperm cryopreservation before RIO,^8^ but previous reports suggest semen cryopreservation remains significantly underutilised with only 24% of patients undertaking this.^3^ In the absence of the preoperative identification of patients with severe oligospermia or azoospermia, it is not possible to identify those patients that would benefit from microsurgical onco‐testicular sperm extraction (micro‐oncoTESE) with simultaneous semen cryopreservation.^9^ The decision to undertake expedited RIO is another practice that potentially compromises fertility outcomes, in spite of the relative lack of evidence to demonstrate any oncological benefit.^8^

The lack of contemporary evidence means that there is a poor understanding of current practices related to fertility assessment and preservation in testicular cancer and of the barriers that lead to clinicians forgoing this aspect of patient care. It may be that clinical practice has not changed to reflect modern cancer referral pathways or chemotherapy outcomes, or that patient related anxiety leads to expedited surgical management. The objective of this study was to assess current international clinician attitudes, practices and barriers towards fertility assessment and preservation in patients undergoing RIO for testicular cancer.

## 2 MATERIALS AND METHODS

This was an international online survey of urologists and urologists in‐training, led by the British Association of Urological Surgeons (BAUS) Sections of Andrology and Oncology and the British Urology Researchers in Surgical Training (BURST) (Appendix S1). It has been reported using the Checklist for Reporting Results of Internet E‐Surveys (Appendix S2). The survey was divided into two components surrounding care at the time of RIO: (i) fertility assessment and preservation and (ii) testicular prosthesis, with the former of these components being the focus of this study.

### 2.1 Design

The survey was obtained using two sampling techniques.^10^ The first was a probability list‐based sampling approach using email addresses to target urologists via national urological societies in the United Kingdom, which included the BAUS and BURST mailing lists. To increase response rates and to reach international urologists, a non‐probability sampling technique was used. This comprised of an unrestricted self‐selected survey method by including a link to the survey on the social media platform Twitter.

### 2.2 Institutional review board approval and informed consent process

The online survey was exempt from requiring ethical approval though informed consent was obtained from the respondents within the survey. The survey was anonymous (unless respondents volunteered their names for acknowledgment purposes) and responses were stored in a password‐protected Research Electronic Data Capture (REDCap) database hosted by University College London (UCL). There was no collection of patient data or the need to access confidential patient records. The access to respondent response data was allocated in advance of study commencement to only three authors (A. E., A. K., and N. B.) in the study group.

### 2.3 Development and pretesting

The REDCap platform was used to deliver the survey and to store responses. The scope, choice of questions and format were drafted by A. E., A. K., and M. S. and then revised by all authors including members of the executive committees of BAUS Andrology and Oncology. The formatting, reliability and functionality of the online REDCap survey were tested in multiple rounds by all authors.

### 2.4 Recruitment process, description of the sample having access to the questionnaire, and survey administration

The online survey was first advertised in February 2021 and kept live for over 3 months from 10/02/2021 to 31/05/2021. The recruitment process primarily used social media and emailing lists as described above. Respondents were eligible to complete the survey if they were a practicing urologist or urologist in‐training that routinely performed RIO for TESTICULAR CANCER. No restrictions were made by country of practice. There were no financial incentives offered for completion.

### 2.5 Preventing multiple entries from the same individual

Respondents were provided with instructions prior to survey completion to outline the requirement for single survey completion only. The REDCap platform ensured that only a single entry could be recorded for each allocated username. Data were also collected on participant clinician grade place of work, as well as the timing of survey completion, which facilitated the identification of potential duplicate records.

### 2.6 Survey content

The full content of the survey is outlined in Appendix S1. The format of questions primarily required the respondent to select the single best or most applicable answer from a short list. The survey consisted of 50 questions. This included data on participant details include

- Geographical location
- Status as fully qualified urologist or urologist in training
- Presence of specialist interest in andrology
- Place of work (secondary or tertiary centre)

The remaining questions made use of a Likert scale to assess clinician practices and attitudes towards fertility assessment and preservation, and timing of RIO. Respondents were able to re‐review and change their answers prior to submitting their final responses.

### 2.7 Survey outcomes

#### 2.7.1 Primary outcome

1. The proportion of urologists who routinely offer semen cryopreservation prior to RIO

#### 2.7.2 Secondary outcomes

1. The categorised reasons for urologists not routinely offering semen cryopreservation prior to RIO
2. The proportion of urologists who perform semen analysis prior to RIO
3. The proportion of urologists who perform testicular serum hormone profile (serum testosterone/luteinising hormone [LH]/follicle‐stimulating hormone [FSH]) prior to RIO
4. The categorised reasons for urologists not undertaking pre‐operative semen analysis and serum hormone profile
5. The proportion of urologists who perform expedited RIO within 48 h of diagnosis.

For the purposes of this survey, ‘offering’ a service was defined as discussing and counselling a patient regarding the availability of an investigation or treatment option, rather than the ability to deliver it at their own institution or ensure financial coverage for this service.

### 2.8 Analysis

In this study we applied descriptive statistical analyses only in Microsoft Excel to determine the key cohort parameters and study outcomes. The use of more complex statistical models was not required in order to answer our primary and secondary endpoints. Although this meant that this study was not powered to answer more nuanced research questions, including predictors of certain behaviours or comparisons between groups, it allowed for an important oversight of current decision‐making processes and attitudes. Where respondents submitted a survey that contained missing data, analysis was still performed if valid responses had been recorded for individual parameters. Where data were missing for individual parameters, then the denominator was changed accordingly.

## 3 RESULTS

### 3.1 Respondent characteristics and response rate

A total of 393 respondents completed the survey; respondents were from six continents and 45 different individual countries (Table 1). The majority of respondents were from the United Kingdom (65.9%), with major contributing continents including Europe (15.8%), Asia (5.1%), South America (3.1%) and Australasia (2.8%) (Table 1). Appendix S3 provides a detailed summary of respondents by individual country, and Appendix S4 provides a breakdown of respondents from the United Kingdom by region.

**TABLE 1:** International respondent characteristics.

International respondent characteristics	*Number*, (%)
Geographical location (*n*, %)	
United Kingdom	259 (65.9)
Europe (mainland)	62 (15.8)
Asia	20 (5.1)
South America	12 (3.1)
Australasia	11 (2.8)
North America	4 (1.0)
Africa	6 (1.5)
Clinician grade (n, %)	
Fully qualified urologist	
Consultant/attending	236 (60.1)
Associate specialist	23 (5.9)
Urologist in training (residents and fellows)	
Urology trainee/resident or specialty doctor	100 (25.5)
Urology fellow	21 (5.3)
Other (international doctor)	13 (3.3)
Affiliated institution (*n*, %)	
Tertiary care (teaching hospital or regional referral centre)	248 (63.1)
Secondary care (district general hospital or community hospital)	143 (36.4)
No response	2 (0.5)
Sub‐specialist interest in andrology	
Yes	109 (27.8)
No	283 (72.2)
No response	1 (0.3)
Individual annual RIO volume (mean cases per year)	
<5	177 (45.1)
5–10	182 (46.4)
>10	33 (8.4)
No response	1 (0.3)

Abbreviation: RIO, radical inguinal orchidectomy.

The majority of respondents were fully qualified urologists (66.0%); working in tertiary care centres (teaching hospitals or regional referral units, 63.1%), but had no sub‐specialist interest in andrology (72.2%). In terms of mean surgical volume, 8.4% (*n* = 33) performed >10 RIOs per year (Table 1).

### 3.2 Outcomes

Of the respondents, 57.1% reported that they would routinely offer semen cryopreservation to all patients undergoing RIO for testicular cancer. Those who were most likely to always offer semen cryopreservation were from Australasia (72.1%; *n* = 8/11); South America (66.6% 8/12); the United Kingdom (61.7%; *n* = 160/259), and Europe (54.8%, *n* = 34/62), rather than North America (25%, *n* = 1/4), Asia (25%; *n* = 5/20), or Africa (16.7%; *n* = 1/6). There did not appear to be any significant difference between tertiary (54.8%) or secondary care centres (60.1%), or between fully qualified urologists (58.9%) and urologists in training (54.8%). Of those who offered semen cryopreservation, 62.5% respondents reported that this would be taken up by >50% of patients.

**FIGURE 1:**
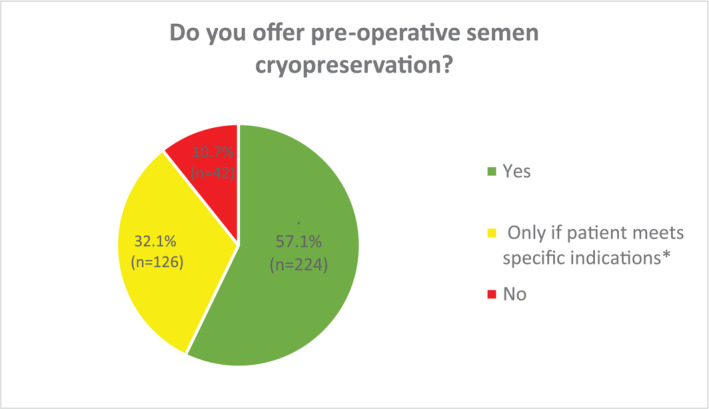
Routine semen cryopreservation in patients undergoing radical inguinal orchidectomy for testicular cancer. *Specific indications: (i) patient had not had children before (fertility potential unknown); (ii) abnormal contralateral testis at clinical assessment pre‐operatively.

**FIGURE 2:**
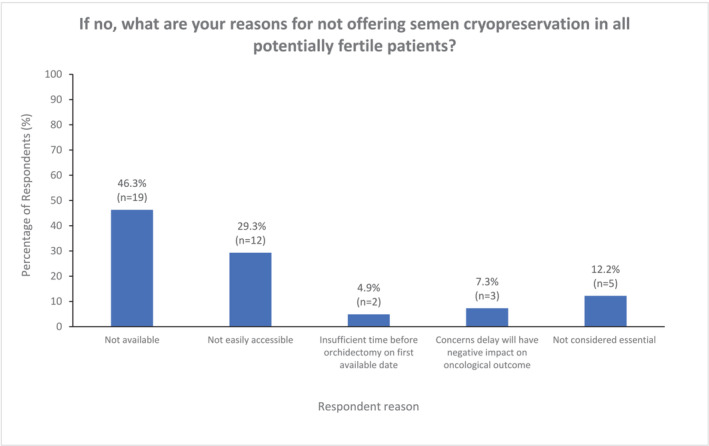
Respondent reasons for not offering semen cryopreservation prior to radical inguinal orchidectomy for testicular cancer (sub‐group analysis of those who do not offer cryopreservation, *n* = 41).

A smaller proportion of participants (32.1%) would only offer semen cryopreservation in specific circumstances where the patient has not had previous children (unknown fertility potential) or there was an abnormal contralateral testis on preoperative examination (Figure 1). 10.7% reported that they did not offer semen cryopreservation (Figure 1). Figure 2 outlines the specific respondent reasons for not offering semen cryopreservation. In the majority of cases this was due either lack of availability (*n* = 19, 46.3%); or limited accessibility (*n* = 12, 29.3%), rather than concerns regarding the need to achieve expedited RIO for oncological reasons. Although it could be assumed that developing nations accounted for a significant proportion of those cases where lack of availability was cited as the reason for not offering semen cryopreservation (Africa: 15.7%, *n* = 3; Asia: 42.1%, *n* = 8; South America: 10.5%, *n* = 2), this was also reported in the United Kingdom (21.1%, *n* = 4), Europe (5.3% *n* = 1), and North America (5.3%, *n* = 1).

**FIGURE 3:**
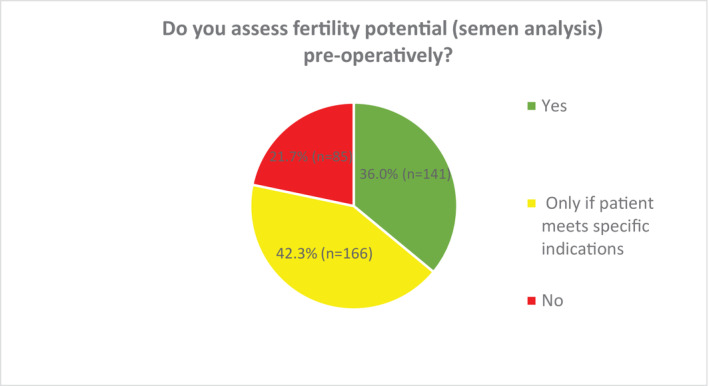
Pre‐operative semen analysis in patients undergoing radical inguinal orchidectomy for testicular cancer. *Specific indications: (i) If the patient has not had children before (ii) If there is an abnormal contralateral testis pre‐op.

**FIGURE 4:**
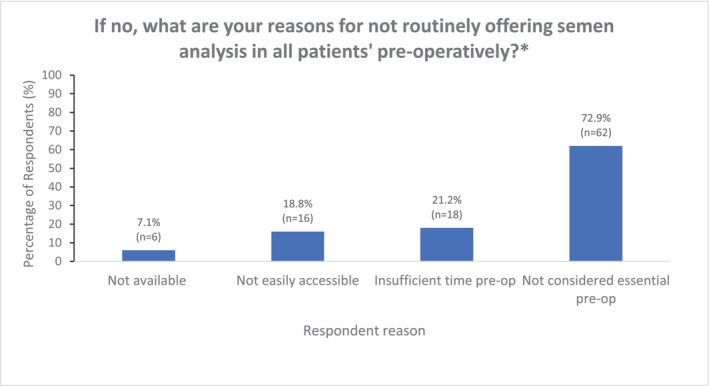
Respondent reasons for not offering semen analysis prior to radical inguinal orchidectomy. (respondents allowed to select *multiple options* that were applicable; respondents; *n* = 85). *Specific indications: (i) If the patient has signs/symptoms of hypogonadism (ii) If there is an abnormal contralateral testis pre‐op.

Figure 3 outlines the proportion of respondents that perform baseline semen analysis prior to RIO; this was performed in a relative minority of cases (36.0%), with the remaining 64.0% either not offering this (21.7%) or only doing so in specific circumstances (no previous children or abnormal contralateral testis) (42.3%). Of those who did not offer pre‐operative semen analysis (*n* = 85), this was primarily because it was not considered essential (73.8%, *n* = 53/85) (Figure 4). Those working in tertiary care were more likely to undertake pre‐operative semen analysis (41.1%; 102/248) than those in secondary care (27.3%; 39/143), as were those with a sub‐specialist interest in andrology (41.2%; 45/109) compared with non‐andrologists (33.9%; 96/283).

**FIGURE 5:**
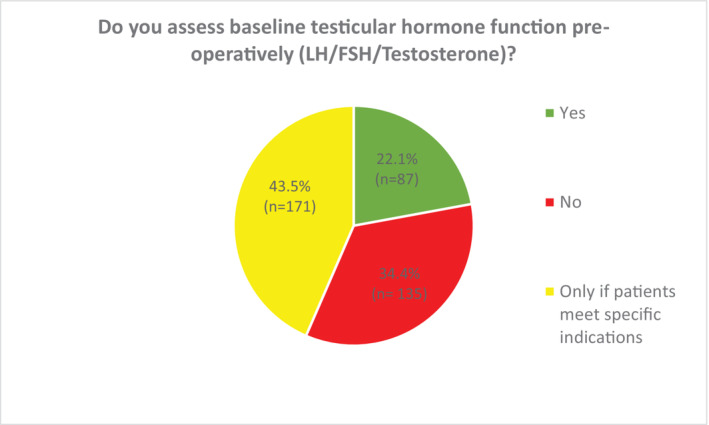
Pre‐operative assessment of testicular hormone function in patients undergoing radical inguinal orchidectomy for testicular cancer.

**FIGURE 6:**
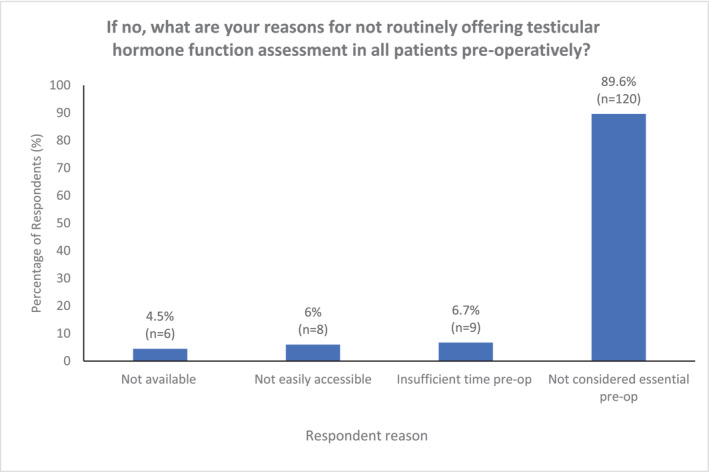
Respondent reasons for not performing preoperative testicular hormone function assessment.

When considering pre‐operative testicular hormone functional assessment (LH, FSH and testosterone), this was routinely offered by a minority of respondents (22.1%) (Figure 5). Of them, 77.9% either did not offer this at all (34.4%), or only performed this in specific circumstances (evidence of hypogonadism or abnormal contralateral testis) (43.5%). In similarity to pre‐operative semen analysis, the majority of those not offering testicular hormonal assessment reported that the reason for forgoing this was because they felt it was not essential (89.6%) (Figure 6). Those working in tertiary care were more likely to undertake pre‐operative testicular hormone functional assessment (27.8%) than those in secondary care (12.5%), as were those with a sub‐specialist interest in andrology (40.4%) compared with non‐andrologists (15.2%).

**FIGURE 7:**
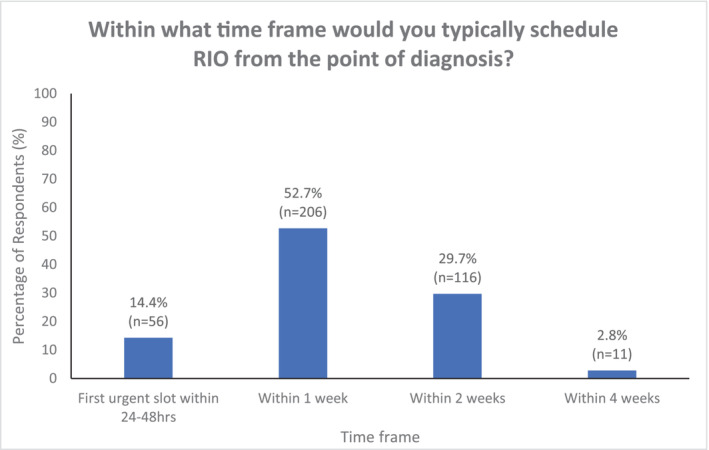
Scheduled timing of radical inguinal orchidectomy from time of diagnosis.

Of the respondents, 14.4% reported that they would offer expedited RIO within 48 h of diagnosis (Figure 7). When considering that a further 52.7% of respondents would perform RIO within 1 week of diagnosis, this meant that only 32.5% would wait more than 1 week to proceed to surgery (Figure 7). Those that performed expedited RIO (≤48 h) were more likely to be from Europe (21.0%), Africa (16.7%) and Asia (15.0%) as to compared with the United Kingdom (12.0%), South America (8.3%) and Australasia (0%). There did not appear to be any significant differences observed between tertiary (13.3%) and secondary centres (14.7%), or andrologists (17.4%) or non‐andrologists (12.7%) when considering expedited (<48 h) RIO. When considering semen cryopreservation, 31.2% felt that no delay to RIO was acceptable to allow for this. Of those respondents that did feel delay was acceptable (68.8%), only 12.3% felt that RIO could be delayed more than 14 days beyond diagnosis to facilitate this. With regard to semen analysis, 61.6% felt that delay to RIO would be acceptable to facilitate this.

## 4 DISCUSSION

This study provides an important international assessment of contemporary urological practice regarding fertility assessment and preservation in testicular cancer patients undergoing RIO. To the best of the authors' knowledge, there are no previous international surveys of urologists, which assess this research question, and as such, these data represent the best available evidence to inform future improvements in fertility practice in this cohort of patients.

In spite of international guideline recommendations,^8^ only 57.1% of urologists routinely offer semen cryopreservation prior to RIO. Although these include data from developing countries where this practice is more likely to be unavailable or unaffordable, the majority of included participants were from developed countries within the United Kingdom and mainland Europe (81.7%). The proportion of urologists in our survey, who routinely offer semen cryopreservation does, however, appears higher than previous surveys of clinical oncologists managing testicular cancer patients; in 2002, Schover et al. reported that 10% of surveyed US oncologists offered semen cryopreservation to all eligible patients while in 2010, Raba et al. reported that 42% of surveyed Arabic oncologists would do so.^10^, ^11^ Although a further 32.1% of urologists in our survey did offer semen cryopreservation if specific indications were met (unknown fertility or abnormal contralateral testis), previous data have demonstrated that clinical examination of the contralateral testis can be a misleading surrogate for fertility and is associated with an average overestimation of testis volume by 5 ml.^12^ Although the proportion of urologists who routinely offer semen cryopreservation may have increased if the survey had also included the delivery of this after RIO, the focus of this survey was to understand the extent of current optimal practice prior to RIO in accordance with international guidelines.^8^

On considering the reasons for not offering semen cryopreservation, the perceived lack of availability or accessibility to semen cryopreservation was a major contributing factor with 75.6% of those not offering semen cryopreservation citing this as the main barrier. Given that respondents from developed countries were included in this response, this suggests that this is not simply attributable to prohibitive cost or healthcare infrastructure but that a lack of streamlined referral pathways or inadequate awareness of local fertility services may also contribute to under‐referral. The relative infrequency of RIO for testicular cancer relative to other urological cancer surgeries (only 8.4% performed >10 per year) may also reduce familiarity with such processes; Schover et al. have previously reported that oncologists who cared for a higher annual volume of eligible testicular cancer patients were more likely to offer semen cryopreservation.^10^

The motivation of urologists to achieve good oncological outcomes and facilitate timely future adjuvant treatment has led to widespread adoption of RIO within 48 h (14.4%) or 1 week (52.7%) of diagnosis, with 31.2% in this survey reporting that no delay to RIO was acceptable to facilitate semen cryopreservation. This is in spite of the absence of any evidence that demonstrates expediting RIO after clinical diagnosis improves oncological outcomes for testicular cancer patients.^13^ In an optimised, tertiary setting, Moody et al. have demonstrated that comprehensive fertility assessment and preservation typically requires 1 week to be achieved.^3^ Although such a comprehensive assessment will not be needed for all patients, existing data demonstrate that approximately 50% of patients are interested in semen cryopreservation when this is offered.^14^ In such patients, forgoing fertility preservation for expedited RIO may compromise fertility outcomes without significant oncological benefit.

Furthermore, Schoever et al. have previously described an important discrepancy between the perceived barriers to semen cryopreservation held by clinicians and patients. Moreover, oncologists were less likely to offer semen cryopreservation due to prohibitive cost, homosexuality, HIV status or the presence of aggressive disease, these issues were not felt to be prohibitive by patients.^10^

The proportion of urologists in this survey who routinely assess pre‐operative semen parameters (36.0%) and hormone profile (22.1%) is even lower than those values seen for semen cryopreservation. In contrast to the barriers reported to semen cryopreservation, there appears to be a strong perception amongst urologists that these do not represent essential investigations. This perception may be influenced by the relative lack of strong recommendations in international guidelines. However, forgoing these investigations means that the opportunity to assess for (i) pre‐operative oligo/azoospermia and perform optimised approaches for simultaneous oncological management and fertility preservation (onco‐microTESE) and (ii) the endocrine function of the testes and any associated hypogonadism, are both entirely overlooked. As described by Moody et al.,^3^ most urologists internationally appear to perform RIO without a detailed functional assessment in a way that would not be done for the surgery of other essential paired organs.

It is important to recognise a number of limitations within this study. The use of global social media platforms, such as ‘X’, formerly known as ‘Twitter’, to advertise and distribute this survey via an openly available link meant that there is a relatively limited understanding of the number of urologists that would have seen, and chosen not to respond to the survey, or indeed the proportion of urologists that do not have access to such platforms. This unfortunately represents a source of selection bias within this study, and it is not possible to accurately provide an overall response rate. The authors do also accept that andrologists or those with an interest in fertility would be more likely to complete this survey, and that it would have been less accessible to non‐English speaking countries.

It is also important to acknowledge the scope of this survey, which has been specific to the practices of semen cryopreservation, semen analysis and hormonal profile investigations, rather than more broad aspects of patient counselling around family planning and fertility aspirations of the individual patient. The survey was focused on RIO for testicular cancer by the urologist, and so the extrapolation of its findings to other urological operations or cancers, or even to the practices of other healthcare professionals who are involved in managing testicular cancer patients, would not be valid.

Although the international breadth of this study acts as a unique strength of the data, this introduces a heterogenous respondent population working in diverse healthcare systems. The authors do accept that given 65.9% of respondents were from the United Kingdom, the findings are likely most closely aligned with practice within the UK National Health Service (NHS). In the UK NHS, semen parameters and hormonal profile investigations can be undertaken without financial charge, and sperm can be stored without charge for 10 years. In contrast to this, there are data to suggest a mean fee in the United States of $358 for cryopreservation, with annual maintenance fee $243, with other wide variations in cost across individual European countries.^15^, ^16^ Future work would include developing an agreed standard of care, understanding why specific barriers exist in various healthcare settings.

Survey not formally validated.

## 5 CONCLUSION

A significant proportion of international urologists do not offer pre‐operative fertility assessment and preservation in men undergoing RIO for testicular cancer. Assessments of fertility potential and testicular function are erroneously often only considered based on clinical examination of the contralateral testis. Surgery is performed in an expedited fashion within 1 week in the majority of patients. Urologists perceive there to be a lack of access and availability to fertility services and that delay to RIO to allow for fertility considerations is often not acceptable. Improvements in fertility outcomes for testicular cancer patients will require these concerns to be addressed through improved clinical training and pathways as well as evidence‐based guideline recommendations.

## AUTHOR CONTRIBUTIONS

Abi Kanthabalan and Anthony Emmanuel constructed the survey. Abi Kanthabalan was the primary author of this paper and conducted the data analysis. Anthony Emmaniel, Cameron Alexander, Nikita Bhatt, Veeru Kasivisvanathan and Maj Shabbir were the reviewers of the article. Maj Shabbir was the lead for this concept and project.

## CONFLICT OF INTEREST STATEMENT

There are no conflicts of interest from the authors within this study.

## Supporting information


**Appendix S1.** Supporting Information
