# Supplementary material for: An international survey of contemporary practices towards fertility assessment and preservation in patients undergoing radical inguinal orchidectomy for testicular cancer
Source: BJUI Compass. 2024 Apr 26;5(5):445–53. doi: 10.1002/bco2.356 (PMC11090774; doi:10.1002/bco2.356)
Supplement: Supplementary file 1 — Appendix S1. Supporting Information [file BCO2-5-445-s001.docx]

Appendix 1


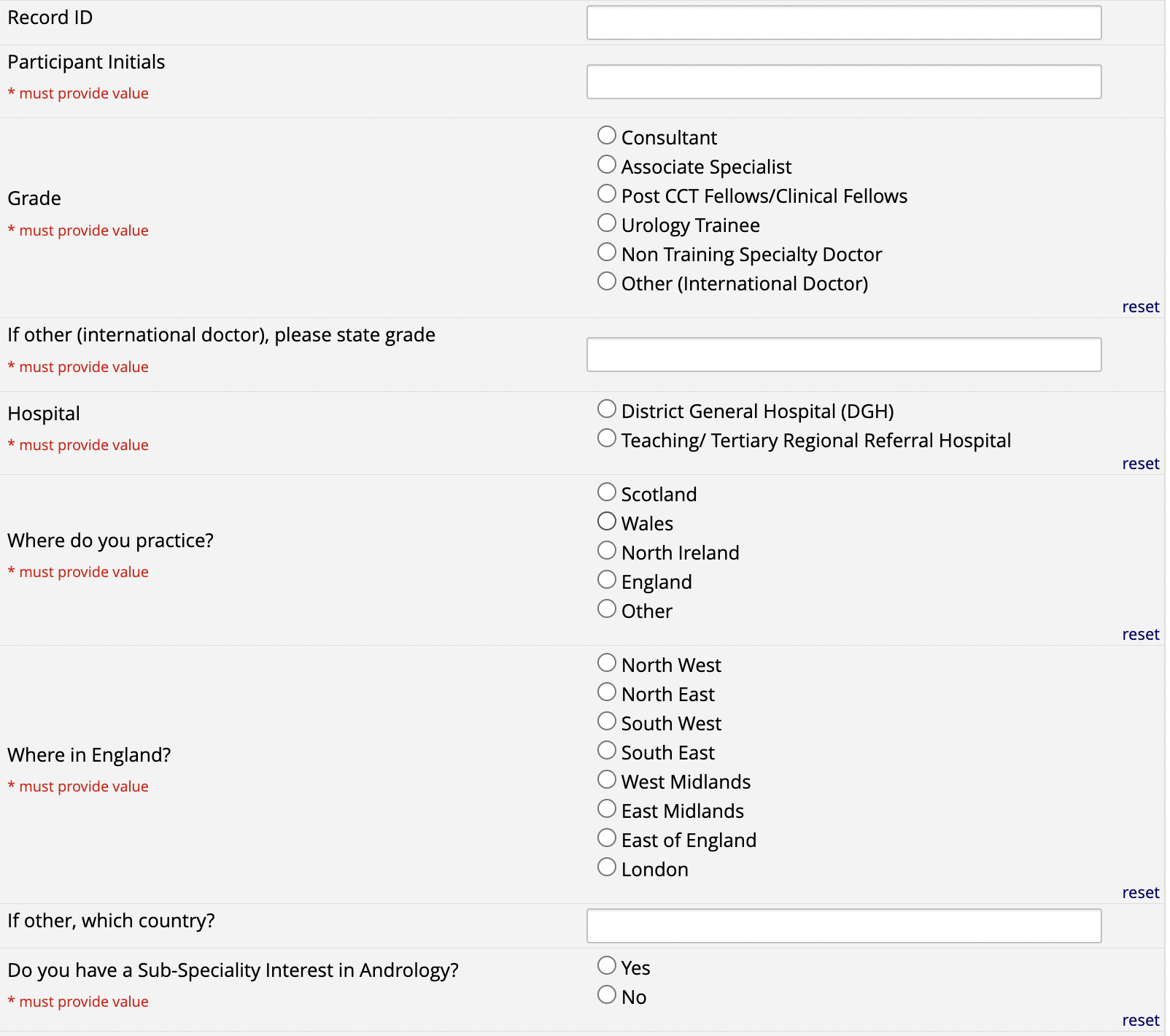


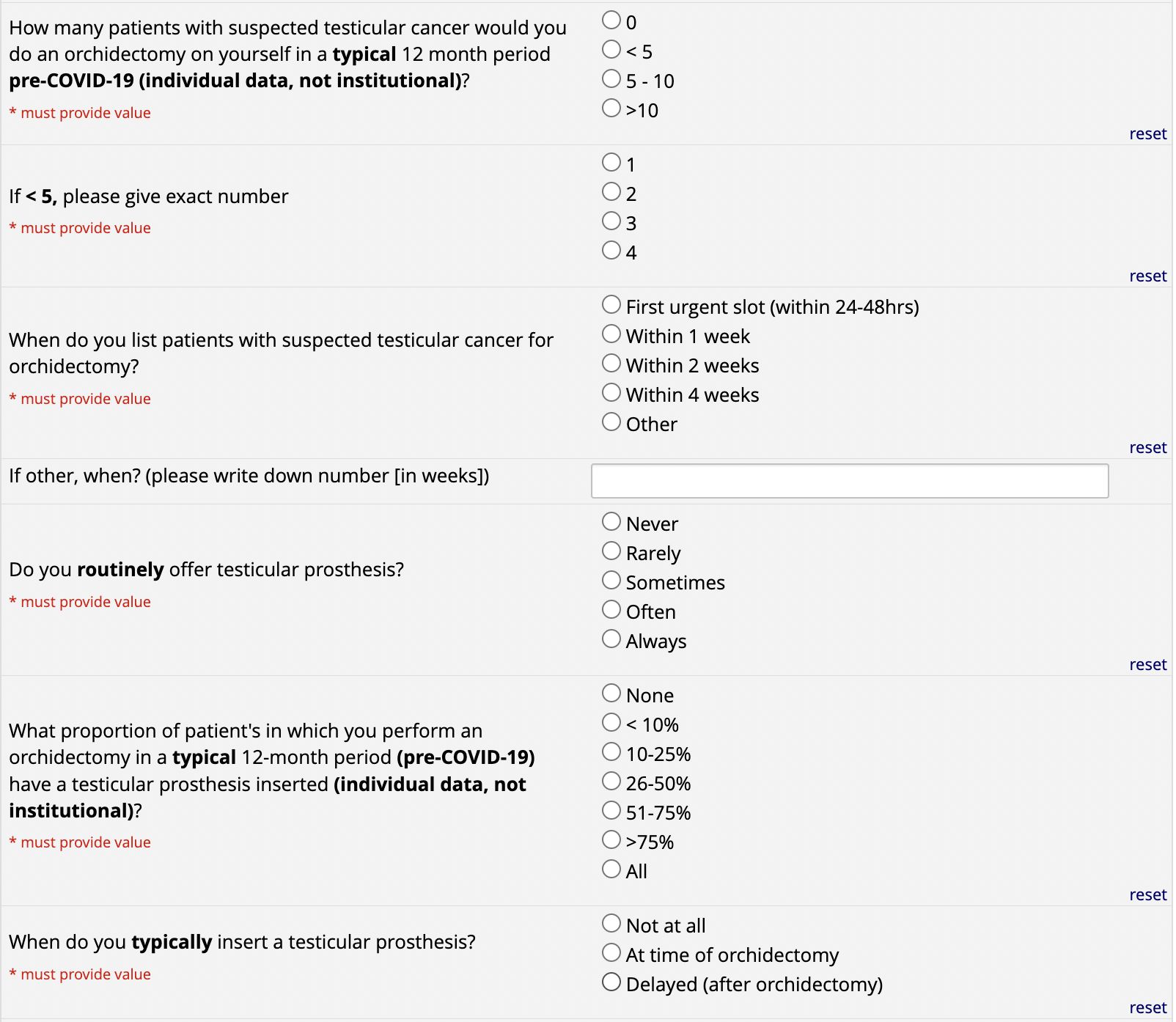

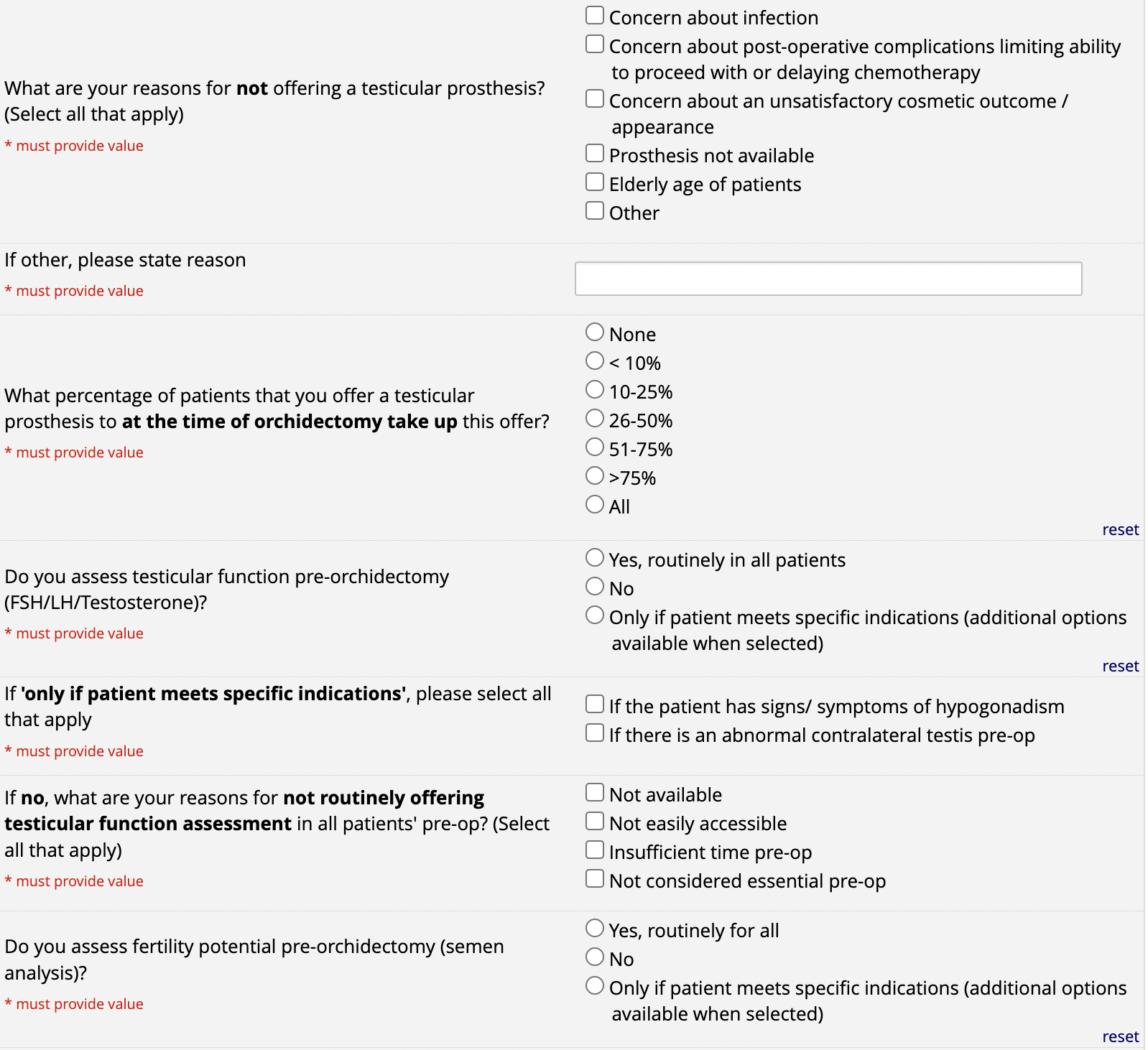

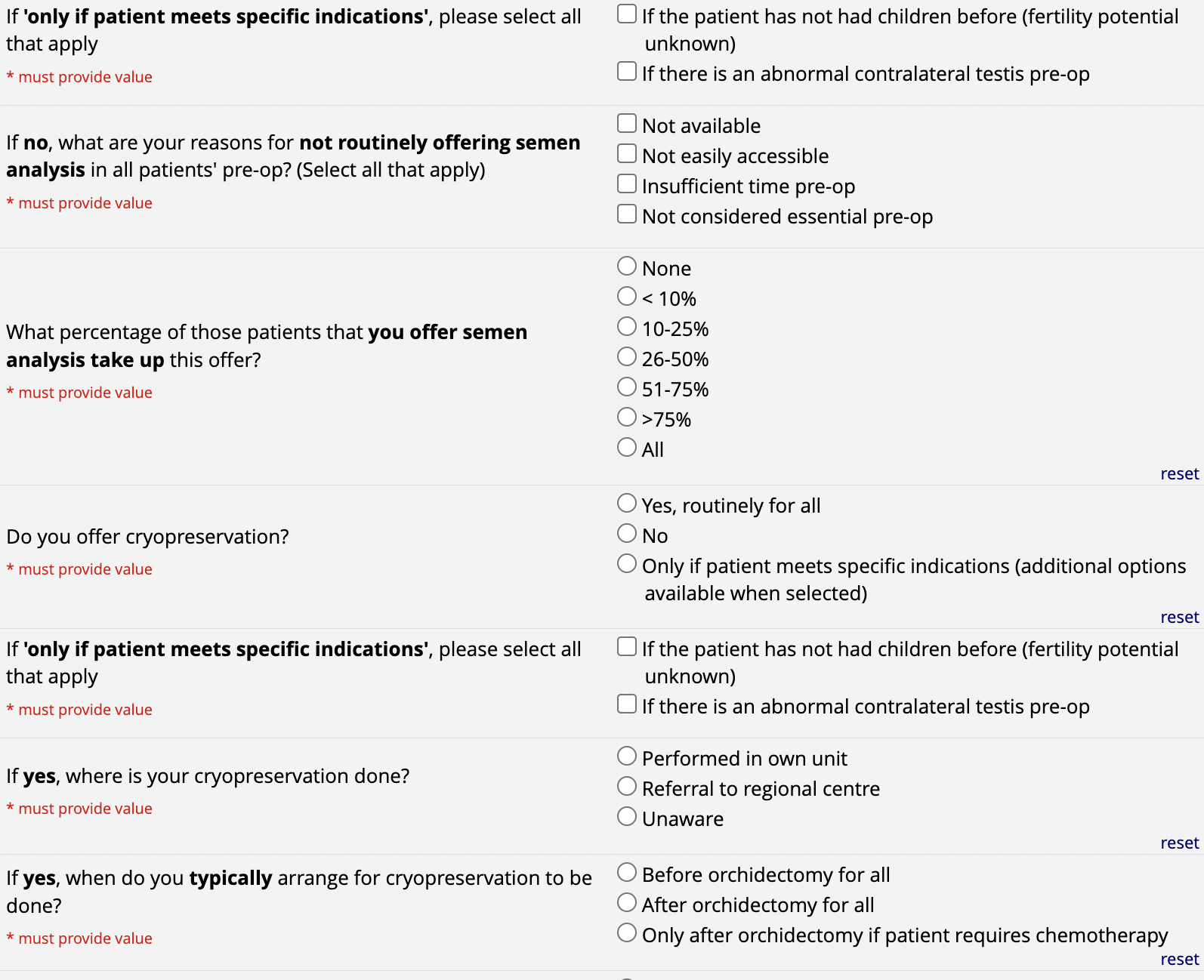

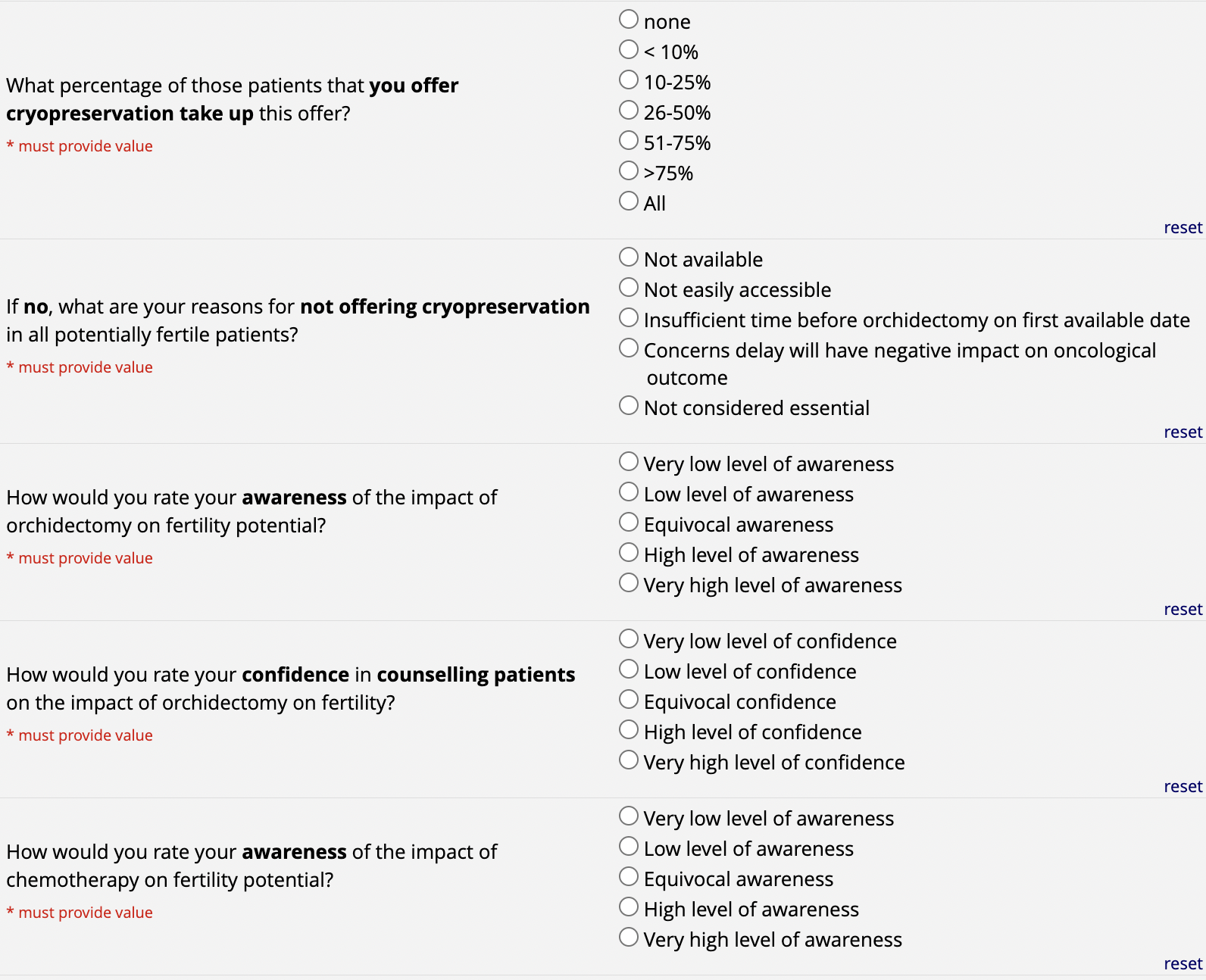

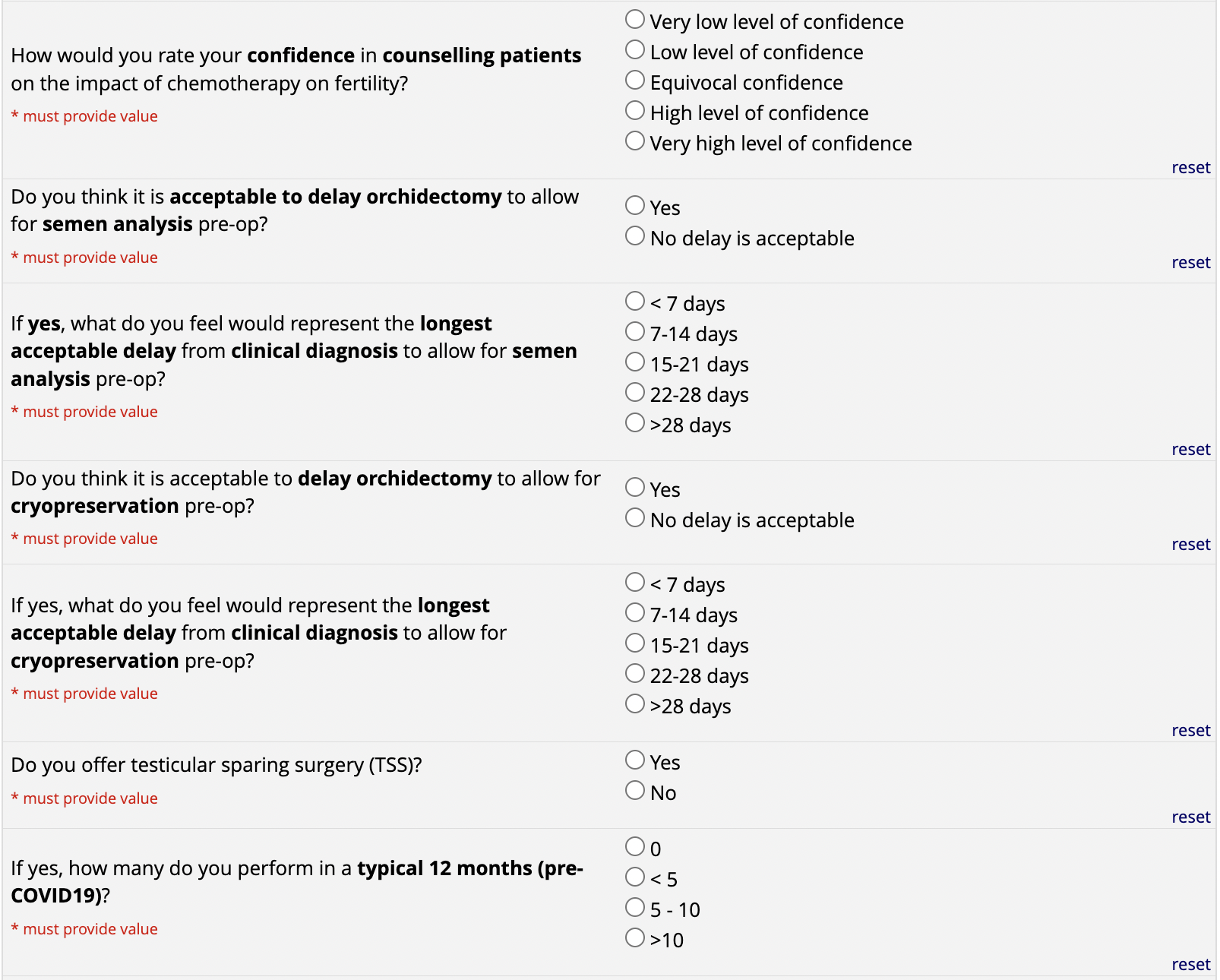

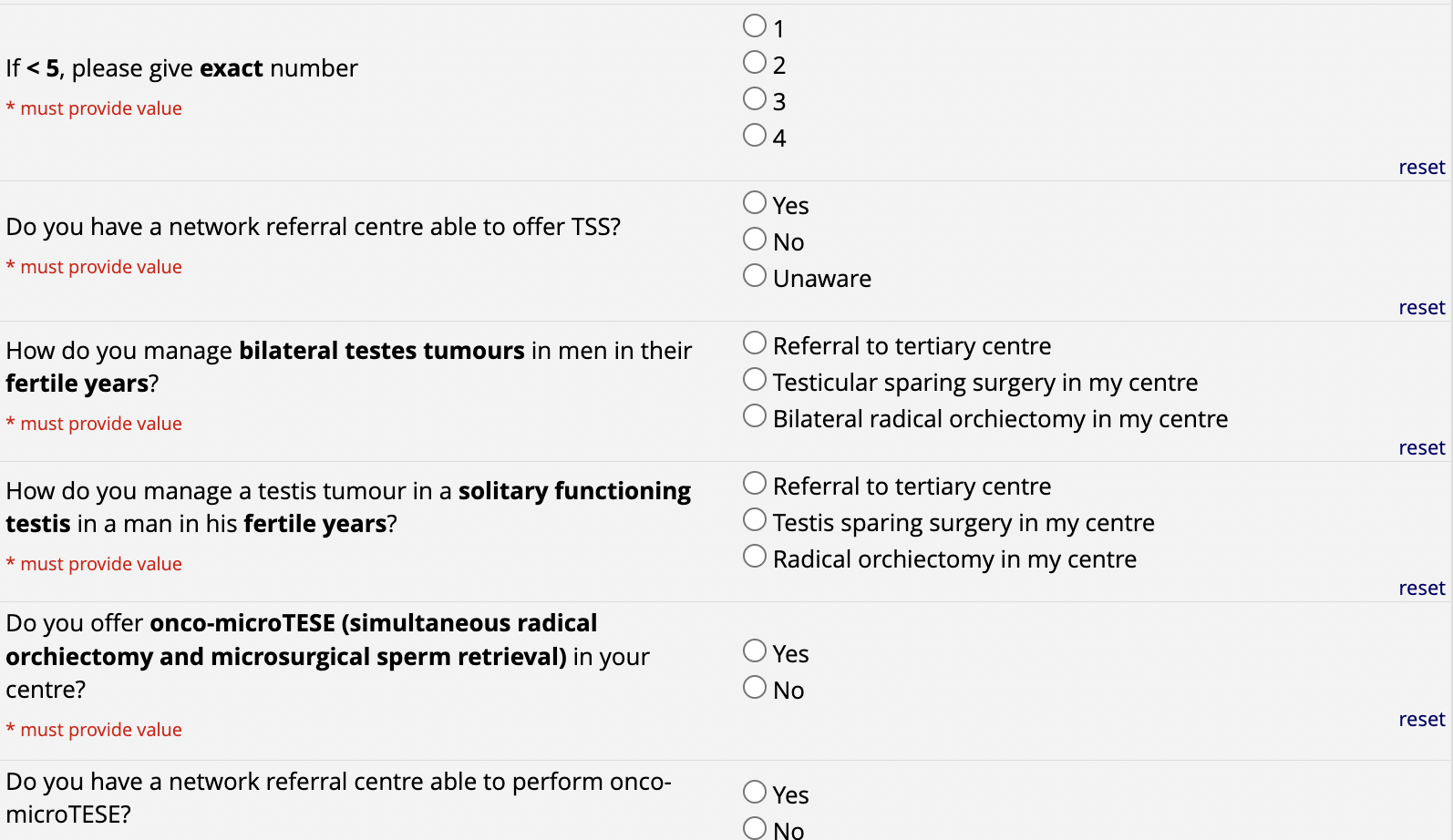


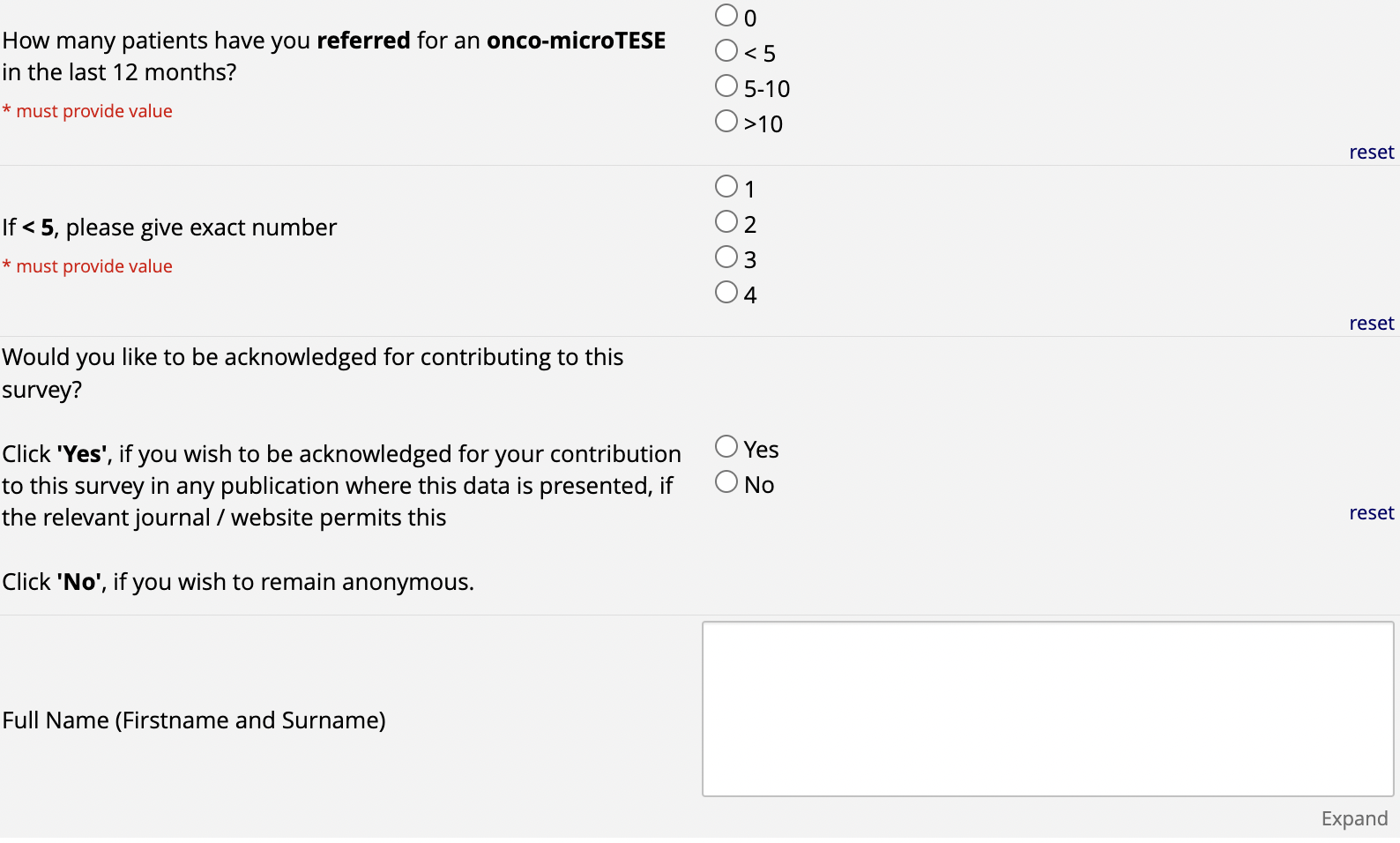


Appendix 2

| **Item Category** | **Checklist Item** | **Explanation** |
| --- | --- | --- |
| **Design** |  |  |
|  | Probability list-based sampling  Non-probability sampling technique | Open survey |
| **IRB (Institutional Review Board) approval and informed consent process** |  |  |
|  | IRB approval | Exempt from requiring ethical approval |
|  | Informed consent | Informed consent was obtained from the respondents within the survey. The survey was anonymous (unless respondents volunteered their names for acknowledgment purposes)  responses were stored in a password protected Research Electronic Data Capture (REDCap) database hosted by University College London (UCL). There was no collection of patient data or the need to access confidential patient records. The access to respondent response data was allocated in advance of study commencement to only three authors |
|  | Data protection | There was no collection of patient data or the need to access confidential patient records. The access to respondent response data was allocated in advance of study commencement to only three authors |
| **Development and pre-testing** |  |  |
|  | Development and testing | The scope, choice of questions and format were drafted by AE, AK, MS, and then revised by all authors. The formatting, reliability and functionality of the online REDCap survey was tested in multiple rounds by all authors |
| **Recruitment process and description of the sample having access to the questionnaire** |  |  |
|  | Open survey versus closed survey | Open survey |
|  | Contact mode | Email link and social media link |
|  | Advertising the survey | Email link and social media link |
| **Survey administration** |  |  |
|  | Web/E-mail | Email link and social media link – link direct to survey and responses recorded in RedCap |
|  | Context | Social media link - Twitter |
|  | Mandatory/voluntary | Voluntary |
|  | Incentives | No incentives |
|  | Time/Date | 10/02/2021 to 31/05/2021 |
|  | Randomization of items or questionnaires | No randomization |
|  | Adaptive questioning | Adaptive questioning was applied using the branching logic in REDCAP |
|  | Number of Items | 48 |
|  | Number of screens (pages) | 3 |
|  | Completeness check | Records were checked for completeness when survey was closed |
|  | Review step | Participants could go back and review answers |
| **Response rates** |  |  |
|  | Unique site visitor | Record ID was created by redcap for each individual participant. Identifiying information was just grade and hospital of each participant |
|  | View rate (Ratio of unique survey visitors/unique site visitors) | We have no way of counting this due to the varied ways of advertising the survey through social media and email |
|  | Participation rate (Ratio of unique visitors who agreed to participate/unique first survey page visitors) | As above |
|  | Completion rate (Ratio of users who finished the survey/users who agreed to participate) | 393 participants took part in this survey |
| **Preventing multiple entries from the same individual** |  |  |
|  | Cookies used | We did not use any cookies |
|  | IP check | We did not use IP address of the client computer to identify potential duplicate entries from the same user |
|  | Log file analysis | We did not use any log file analysis. |
|  | Registration | Participants only had to register and complete survey once |
| **Analysis** |  |  |
|  | Handling of incomplete questionnaires | Participants could complete any number of questions. If there was no answer given, this response was completed from sub analysis |
|  | Questionnaires submitted with an atypical timestamp | No timeframe |
|  | Statistical correction | There was not any use propensity scores or weighting of items |

Appendix 3

Appendix 4:

| **UK Country** | **Participants** |
| --- | --- |
| **England**  London  North West  North East  South West  South East  West Midlands  East Midlands  East of England | 219 (55.9)  55 (25.1)  29 (13.2)  29 (13.2)  25 (11.4)  32 (14.6)  25 (11.4)  11 (5.0)  13 (5.9) |
| **Scotland** | 27 (6.9) |
| **Wales** | 11 (2.8) |
| **Northern Ireland** | 2 (0.5) |
